# Complete Genome Sequences of Monkeypox Virus from a French Clinical Sample and the Corresponding Isolated Strain, Obtained Using Nanopore Sequencing

**DOI:** 10.1128/mra.00009-23

**Published:** 2023-03-27

**Authors:** Charlotte Balière, Véronique Hourdel, Aurelia Kwasiborski, Quentin Grassin, Maxence Feher, Damien Hoinard, Jessica Vanhomwegen, Fabien Taieb, Paul-Henri Consigny, Jean-Claude Manuguerra, India Leclercq, Christophe Batéjat, Valérie Caro

**Affiliations:** a Institut Pasteur, Université Paris Cité, Environment and Infectious Risks Unit, Laboratory for Urgent Response to Biological Threats (CIBU), Paris, France; b Institut Pasteur, Medical Center (CMIP), Paris, France; DOE Joint Genome Institute

## Abstract

We report the whole-genome sequences of a monkeypox virus from the skin lesion of a French patient and the corresponding isolated viral strain. Both viral genomic sequences were successfully obtained by applying shotgun metagenomics using the Oxford Nanopore Technologies sequencing approach.

## ANNOUNCEMENT

Mpox, an emerging disease of zoonotic origin, is caused by *Monkeypox virus* (MPXV), belonging to the genus *Orthopoxvirus* of the family *Poxviridae*. Traditionally endemic to Western and Central African countries, since May 2022, MPXV has caused the largest multicountry outbreak described so far (1).

As of 3 January 2023, a total of 83,974 confirmed monkeypox cases and 75 deaths had been reported to the World Health Organization, with 4,114 cases in France (2). MPXV harbors a large double-stranded DNA (dsDNA) genome of around 200 kb (3), which has already been fully sequenced using the MinION device (4). Here, as part of a global MPX surveillance effort, we directly sequenced a French clinical sample and the corresponding isolated strain.

A swab was collected from a pustular lesion on the leg of a 36-year-old French man who presented for an infectious disease consultation at the Medical Center, Institut Pasteur, in June 2022. Vero E6 cells were inoculated with the clinical specimen and incubated for 3 days, and the culture supernatant was harvested and tested for the presence of MPXV by PCR. The clinical sample and the corresponding isolate were found positive with respective threshold cycle values of 20 and 15, using a generic MPXV real-time PCR assay (G2R_G), as previously described (5). The lab investigations described in this article were carried out in accordance with the General Data Protection Regulation (regulation [EU] 2016/679 and directive 95/46/EC) and the French data protection law (law 78-17 on 6 January 1978 and decree 2019-536 on 29 May 2019), which do not require a review by an ethics committee for the secondary use of samples collected for healthcare purposes. In such cases, secondary use for research is authorized, as the persons have been informed of such secondary use (article L.1211-2 of the French public health code).

Genomic DNA (gDNA) was extracted from 150 μL each of the clinical sample (CIBU-220035) and isolate (CMIP-P0) using the NucleoSpin Dx virus kit (Macherey-Nagel). Using the Qubit dsDNA high-sensitivity assay kit (Invitrogen), the concentration of gDNA was estimated at 0.261 ng/μL and 6.18 ng/μL for the clinical sample and the isolated strain, respectively.

For Nanopore sequencing, libraries were prepared using the rapid PCR barcoding kit (SQK-RPB004; Oxford Nanopore Technologies). The library for CMIP-P0 was prepared from 5 ng of gDNA, but due to the low gDNA concentration of the CIBU-220035 sample, its library was prepared from the maximal volume input (i.e., 3 μL). Each library was loaded independently onto an R9.4 flow cell (FLO-MIN106) and sequenced on an Mk1C device for 24 h, yielding reads with a Phred quality score of 8. Default parameters were used for all software. MinKNOW v19.12.2 software was used to collect the raw data and the Guppy v3.4 tool to perform base calling. Subsequently, the “What’s in My Pot?” (WIMP) workflow (FASTQ [human+viral] v2021.11.26) was launched for real-time species classification, allowing the assignation of 0.46% and 18.5% of reads to monkeypox virus strain Zaire-96-I-16 (NCBI taxonomy ID 619591) for CIBU-220035 and CMIP-P0, respectively.

Using Minimap2 v2.17-r941 (6), Medaka v1.0.3, and bcftools v1.10.2, a consensus sequence of 197,205 bp was generated for CIBU-220035 and CMIP-P0 by mapping the reads to the reference genome found under GenBank accession number MT903345, with a high coverage depth along the total length (Table 1), and manually curating them using Tablet v1.21 (7). Both viral genomic sequences belonged to lineage B.1 and clade IIb, according to Nextclade (https://clades.nextstrain.org) (8). Comparison of the genomic sequences obtained for the clinical specimen and its respective isolate showed 99% nucleotide identity, with 5 nucleotide substitutions, resulting in 1 amino acid change (a G-to-A change at position 193366 [G193366A] encoded by the ankyrin-like gene) compared to the reference genome MT903345. Despite the limitations of the genome quality obtained using MinION, this result would suggest that the culture on Vero E6 cells induced some molecular changes, immediately after the primary viral isolation.

**TABLE 1:** Nanopore sequencing data for CIBU-220035 and CMIP-P0

Feature	Data for strain:	
	CIBU-220035	CMIP-P0
Run data (Gb)	94.5	96.05
No. of total reads (million)	3.66	3.92
No. of mapped reads (million)	13,597	511,657
Mean length of reads (bp)	2,158	1,814
Mean quality score	11.99	11.68
Avg depth (×)	145	4,456
Avg coverage 20× (%)	99.6	99.9
Consensus sequence length (bp)	197,205	197,205
GC content (%)	33	33

We report the successful and rapid sequencing of MPXV from a pustular lesion specimen and its corresponding strain using a direct sequencing approach with the Nanopore platform, despite the low-viral load sample.

### Data availability.

The consensus genome sequences were deposited in the GISAID EpiPox database (accession numbers EPI_ISL_16260351 and EPI_ISL_16260402) and at GenBank (accession numbers OQ249660 and OQ249661). The raw reads were deposited in the NCBI Sequence Read Archive under accession number PRJNA918303.
